# Value of metabolic parameters in distinguishing primary mediastinal lymphomas from thymic epithelial tumors

**DOI:** 10.20892/j.issn.2095-3941.2019.0428

**Published:** 2020-05-15

**Authors:** Lei Zhu, Xiaofeng Li, Jian Wang, Qiang Fu, Jianjing Liu, Wenchao Ma, Wengui Xu, Wei Chen

**Affiliations:** ^1^Department of Molecular Imaging and Nuclear Medicine, Tianjin Medical University Cancer Institute and Hospital; National Clinical Research Center for Cancer; Key Laboratory of Cancer Prevention and Therapy, Tianjin; Tianjin’s Clinical Research Center for Cancer, Tianjin 300060, China

**Keywords:** FDG PET-CT, lymphoma, metabolic tumor burden, quantitative evaluation, thymic epithelial tumors

## Abstract

**Objective:** A high rate of unnecessary thymectomies has been reported. This study aimed to distinguish primary mediastinal lymphomas (PMLs) from thymic epithelial tumors (TETs) by evaluating volumetric and metabolic parameters with ^18^F-FDG PET/CT.

**Methods:** A total of 136 patients who were pathologically diagnosed with TETs or PMLs were enrolled, and ^18^F-FDG PET/CT was performed before therapy. Volumetric parameters, including the mean SUV (SUVmean), metabolic tumor volume (MTV), total lesion glycolysis (TLG), and SUVmax, were determined and compared between the 2 subtypes. The diagnostic performance of these parameters was evaluated with receiver operating characteristic (ROC) curve analysis.

**Results:** All parameters significantly differed between patients with PMLs and TETs. Patients with lymphomas were younger and had higher SUVmean, SUVmax, TLG, and MTV values than patients with TETs. The MTV and TLG values had similar diagnostic performance. ROC analysis indicated that the areas under the curves of the SUVmean and SUVmax values performed similarly (approximately 0.76) in differentiating patients with PMLs from TETs, and both values were better than the MTV and TLG values. When age was included with the SUVmax in differentiating TETs from PMLs, the AUC was 0.91, and the sensitivity and specificity increased to 80% and 93%, respectively.

**Conclusions:** The SUVmax and volumetric parameters of ^18^F-FDG PET/CT can be used to distinguish patients with PMLs versus TETs, and thus may aid in preventing unnecessary thymectomies or other invasive operations.

## Introduction

The International Thymic Malignancy Interest Group (ITMIG) has developed a classification system for mediastinal compartments to be used for cross-sectional imaging of mediastinal disease; this system is important for accurate localization, characterization, and differential diagnosis^1,2^. According to the ITMIG standards, the most common primary lesions in the pre-vascular compartment are thymic epithelial tumors (TETs), followed by primary mediastinal lymphomas (PMLs), germ cell neoplasms, and intrathoracic goiters. Therapies for diseases located in the pre-vascular mediastinum markedly differ^3,4^: TETs are usually surgically removed, whereas lymphomas are generally treated with chemotherapy^5^, and asymptomatic, small benign thymic lesions are usually left untreated. Unfortunately, high rates of unnecessary thymectomies or invasive surgical procedures have been reported, owing to misdiagnosis of imaging findings pre-operatively^6,7^. PMLs are the malignancy most likely to be confused with TETs before treatment.

Diversity in clinical management requires accurate diagnosis and thorough evaluation before treatment decisions are made. Traditional imaging modalities, including computed tomography (CT) and magnetic resonance imaging (MRI), have been widely used in clinical practice to evaluate pre-vascular mediastinal neoplasms. Imaging characteristics, including the location relative to the midline, shape, homogeneity of attenuation, adjacent invasion, and coexistent lymphadenopathy, are considered the key evaluation standards for diagnosis and further therapy^8–11^. Shortcomings in morphologic assessment have been reported to lead to misinterpretation, thereby potentially resulting in ineffective or inaccurate management and therapy^6^. In the past decade, ^18^F-fluorodeoxyglucose (FDG) positron emission tomography (PET)/CT has been routinely used to non-invasively identify cancer in early stages, assess metabolic changes in lesions, and simultaneously evaluate the whole-body response to tumors *in vivo*^12^.

Studies have revealed the usefulness of ^18^F-FDG PET/CT in grading TETs by using SUVmax^13–17^; however, few studies have focused on volumetric parameters, including the mean SUV (SUVmean), metabolic tumor volume (MTV), and total lesion glycolysis (TLG) of pre-vascular compartment mediastinal tumors. Furthermore, little is known regarding whether these volumetric parameters can distinguish TETs from PMLs. In this study, our aims were (1) to quantitatively assess the ability of ^18^F-FDG PET/CT volumetric and metabolic parameters to differentiate TETs from PMLs, to avoid non-therapeutic thymectomies, and (2) to compare the diagnostic ability of volumetric and metabolic parameters in the 2 most common mediastinal malignancies of the pre-vascular compartment.

## Materials and methods

### Patient selection

A total of 136 patients with primary thymic lesions who were treated in our institute and hospital between January 2014 and December 2018 were retrospectively reviewed. All patients received a PET/CT examination before an invasive biopsy was performed or therapy was initiated. Some patients were asymptomatic, and the thymic lesions were incidental findings. Patients with mediastinal metastases, lung cancer, or other systemic disorders involving the mediastinum were excluded. All enrolled patients were pathologically diagnosed with primary thymic neoplasms on the basis of surgical findings or core needle biopsy specimens and immunohistochemistry testing. Each patient was required to sign an informed consent form before the PET/CT examination. All procedures involving human participants in this study were approved by the Institutional Research Committee and performed in accordance with ethical standards.

### Molecular imaging acquisition and analysis

All ^18^F-FDG PET/CT scans were performed with a 64 multislice-detector PET/CT scanner (Discovery^ST^; General Electric Healthcare, Waukesha, WI, USA). Patients were fasted 4–6 h before the examination. The blood glucose levels were determined and controlled at < 140 mg/dL before the administration of ^18^F-FDG (3.7 MBq per kg body weight). The protocol included an initial CT scan (120 kV, 100 mA, and a slice thickness of 5 mm). PET images from the head to the mid-thigh were acquired in 3-dimensional mode without breath-holding, with an acquisition time of 2 min per bed position (for a total of 6–8 bed positions) after CT scanning. The CT-based, attenuation-corrected PET images were reconstructed with an iterative algorithm. The attenuation-corrected PET, CT, and fused PET/CT results were reviewed and analyzed by 2 nuclear medicine physicians with 5 and 10 years of diagnostic experience in diagnosis. The mass in the supra-anterior mediastinum was selected as the region of interest. The volume-based parameters, including MTV, SUVmax, peak SUV (SUVpeak), and mean SUV (SUVmean), were obtained with PET VCAR, the semi-quantitative software of the GE workstation. The estimated threshold was 42% of the maximum value. TLG was automatically calculated with MTV multiples of the SUVmean.

### Statistical analysis

SPSS 19.0 was used for statistical analyses and graphing. Group differences of volume-based parameters in each pathologic subtype were compared with analysis of variance for normally distributed data, and the Tukey comparison was further performed as a *post hoc* test. If the variables were not normally distributed, the Kruskal-Wallis test or Mann-Whitney U test was performed instead of analysis of variance or t-test. Interrelations between variables were determined with Pearson correlation analysis. The diagnostic performance of metabolic parameters was evaluated with the area under the curve (AUC) and receiver operating characteristic (ROC) curve. A cut-off value was determined, and the sensitivity, specificity, and accuracy were calculated at a single point on the ROC curve. Generally, the level of statistical significance was set at *P* < 0.05 (two-tailed).

## Results

**Figure 1:**
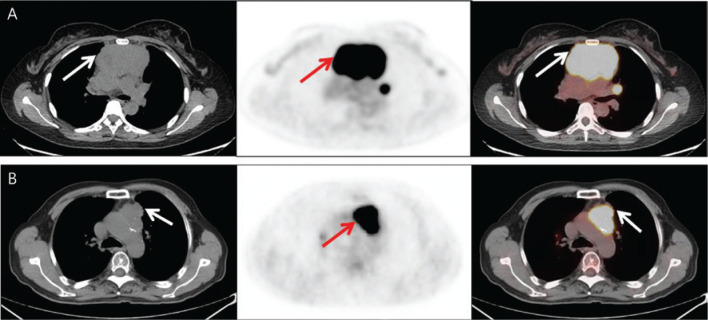
Axial CT, PET, and fusion images (from left to right columns) of examples of primary mediastinal lymphomas (arrows) and thymic epithelial tumors (arrows). Panel A: 43-year-old female patient with diffuse large B-cell lymphoma (CT value = 4–38 Hu; SUVmax = 23.8 g/mL, MTV = 218.0 cm^3^, and TLG = 2774.9 g/mL cm^3^); panel B: 70-year-old male patient with squamous cell carcinoma (CT value = 39 Hu with scattered calcifications; SUVmax = 18.7 g/mL, MTV = 36.28 cm^3^, and TLG = 428.39 g/mL cm^3^).

A total of 136 patients (mean = 41.57 ± 19.12 years, range = 3–77 years) with 71 PMLs and 65 TETs were included in this study. According to the histopathologic results, the patients were divided into the following subgroups: (1) lymphoma, including the most common subtypes (diffuse large B-cell lymphoma, T-lymphoblastic lymphoma, and classic Hodgkin lymphoma) and uncommon types (follicular B-cell lymphoma and anaplastic large cell lymphoma), and (2) epithelial tumors, including all types of thymoma (A-B3) and thymic carcinomas (**Table 1**, **Figure 1**).

**Figure 2:**
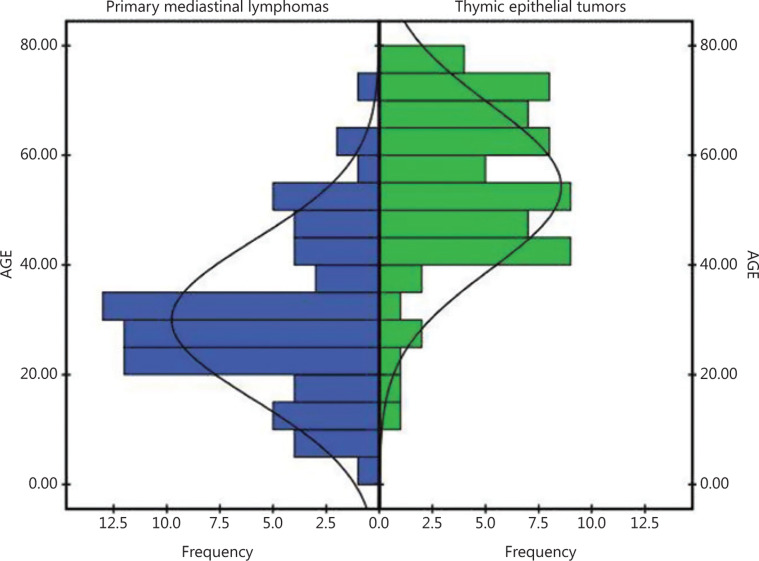
Histograms of age distribution of the patients in this study, showing that most patients with primary mediastinal lymphomas (left panel) were < 40 years of age (76.1%), whereas most patients with thymic epithelial tumors (right panel) were > 40 years of age (87.7%).

In this study, we included 62 male patients (45.6%), but there were differences in sex ratios between groups; males were the majority in the TET group, and females were the majority in the PML group (**Table 1**). The age at the time of diagnosis significantly differed between the 2 subgroups (χ^2^ = 55.19, *P* < 0.001). Patients with PMLs were significantly younger than patients with TETs (30.25 ± 14.44 years *vs.* 54.23 ± 15.16 years; **Table 2**). The histogram in **Figure 2** shows that the age was distributed separately in the 2 groups.

**Figure 3:**
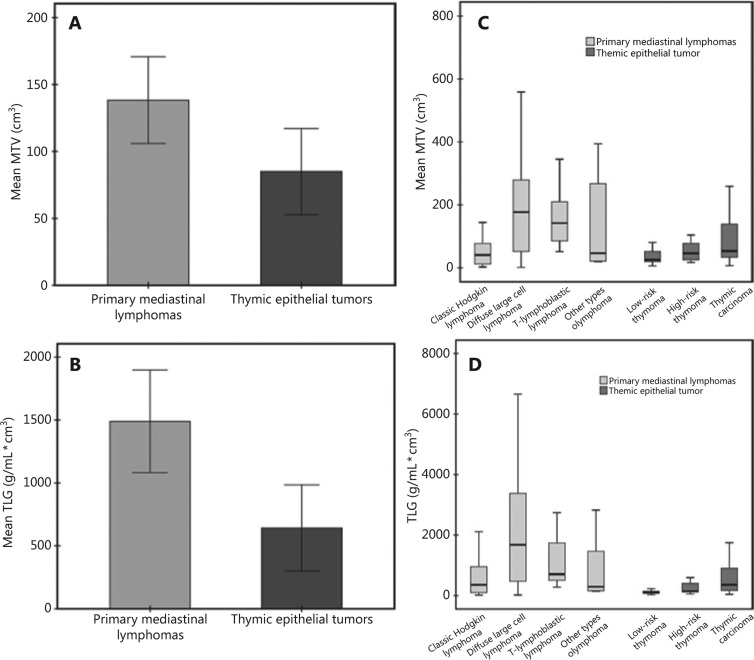
The mean metabolic tumor volume (A) and total lesion glycolysis (B) in the patients with primary mediastinal lymphomas and thymic epithelial tumors, and a separate comparison for each subgroup of primary mediastinal lymphoma (C) and thymic epithelial tumors (D).

SUVmax, the most usable parameter in clinical practice, was significantly higher in the PML group than the TET group, as was the SUVmean. The metabolic tumor burden significantly differed between the subgroups, including MTV and TLG (χ^2^ = 5.74 and χ^2^ = 14.55, respectively; *P* < 0.01). The patients with PMLs had a higher MTV (143.98 ± 149.93 cm^3^) and TLG (1546.2 ± 1838.69 g/mL cm^3^) than patients with TETs (84.99 ± 130.10 cm^3^ and 641.78 ± 1381.45 g/mL cm^3^; **Figure 3A and 3B**, **Table 2**).

Within the PML subgroup, the SUVmax, SUVmean, MTV, and TLG values varied by molecular subtype (*P* < 0.01, **Table 3**). The patients with diffuse large B-cell lymphomas had the highest SUVmax, MTV, and TLG values, followed by those with T-lymphoblastic and Hodgkin lymphomas (**Figure 3C and 3D**). According to the WHO histological classification criteria, the TETs were sub-classified into low-risk (types A, AB, and B1) and high-risk (types B2 and B3) thymomas, and thymic carcinomas. The metabolic and volumetric parameters significantly differed within these subgroups (**Figure 3C and 3D**). Patients with squamous cell carcinoma had the highest SUVmax within the subtypes of epithelial tumors (*P* < 0.01, **Table 3**).

**Figure 4:**
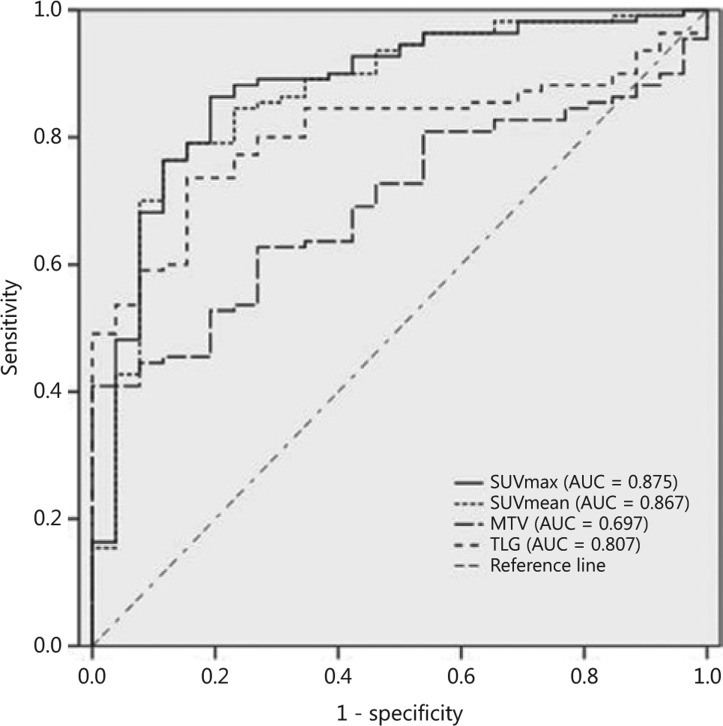
ROC curve and area under the curve of the maximum standard uptake value (SUVmax), mean standard uptake value (SUVmean), metabolic tumor volume (MTV), and total lesion glycolysis (TLG) in differentiating patients with malignant tumors from patients with benign tumors in the pre-vascular compartment of the mediastinum.

The SUVmax, SUVmean, TLG, and MTV values were all able to differentiate malignant tumors from benign lesions with an AUC of 0.875 (95% CI = 0.797–0.953), 0.867 (95% CI = 0.786–0.947), 0.697 (95% CI = 0.606–0.788), and 0.807 (95% CI = 0.732–0.881), respectively (**Figure 4**).

**Figure 5:**
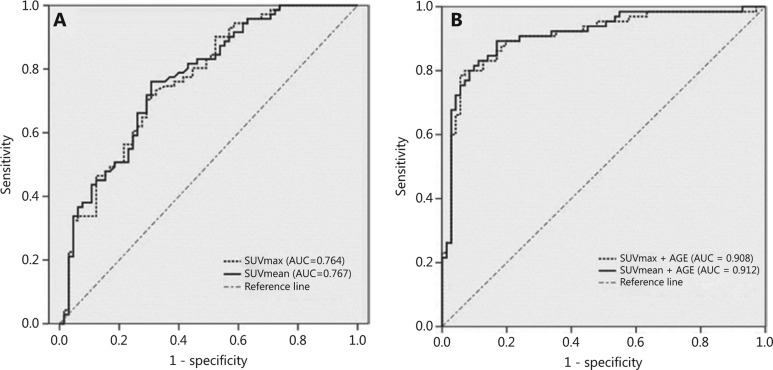
ROC curve and area under the curve of the maximum standard uptake value and mean standard uptake value in differentiating patients with thymic epithelial tumors from patients with primary mediastinal lymphomas in the pre-vascular compartment of the mediastinum (A). The areas under the curve were significantly greater when age was involved as an index (B) in differentiating patients with thymic epithelial tumors from patients with primary mediastinal lymphomas.

In differentiating lymphomas from TETs, the SUVmean had better performance than the other metabolic and volumetric parameters, with the highest sensitivity (76.1%), accuracy (72.8%), and AUC (0.767). With a cut-off value of 12.3, the SUVmax showed slightly higher specificity (70.8%), but lower sensitivity (70.4%), accuracy (70.6%), and AUC (0.764) than the SUVmean (**Table 4**, **Figure 5A**). The TLG value also was able to differentiate patients with PMLs from patients with TETs (AUC = 0.69, 95% CI = 0.599–0.780); the sensitivity, specificity, and accuracy were 70.4%, 63.1%, and 69%, respectively, with a cut-off value of 350.3 g/mL cm^3^. MTV showed the highest specificity (81.5%), but the lowest sensitivity (46.5%) and AUC (0.619). Combining the TLG and SUVmax values had little effect on diagnostic ability; the sensitivity decreased, but the specificity slightly increased to 73.2% (AUC = 0.768, 95% CI = 0.689–0.847; **Table 4**). Both the sensitivity and specificity significantly increased to 80% and 93%, respectively, when age and the SUVmax were combined as an independent tool, thus yielding an AUC of 0.908 (0.855–0.961; **Figure 5B**).

## Discussion

A total of 44% of thymectomies have been reported to be unnecessary or non-therapeutic as a result of misdiagnosis of lymphomas, thymic hyperplasia, cysts, and other benign thymic lesions as thymomas, according to CT scans^6^. Lymphomas account for more than 50% of misdiagnosed cases; thus, pre-operative discrimination of lymphomas from the other anterior mediastinal tumors according to morphologic and functional information is useful. Benign tumors can usually be easily distinguished from malignant tumors in the pre-vascular part of the mediastinum, traditionally on the basis of morphologic features, including the shape, density, heterogeneity, extent of contrast enhancement, and involvement of adjacent tissues or lymph nodes^17,18^. Our study showed that the metabolic tumor burden, including the MTV and TLG, as well as other ^18^F-FDG uptake parameters, such as SUVmax and SUVmean, can be used for selective diagnosis of lymphomas.

PET/CT improves the sensitivity and specificity of diagnosis by providing both anatomic and metabolic information. The SUV, a PET/CT semi-quantitative index demonstrating the uptake of glucose in tumors and normal tissues, has been widely accepted by physicians for daily use. Studies have reported that the SUVmax can help establish differential diagnosis of tumors in the pre-vascular compartment^12–14^, and the SUVmax has also been found to be useful for the differential diagnosis of low- and high-risk TETs^15–17^. Nevertheless, these findings remain questionable because the semi-quantitative SUVmax is the SUV on the highest image pixel reflecting a single-pixel value of the maximum intensity of ^18^F-FDG activity in the tumor, thus ignoring the extent of metabolic abnormalities and differences in tracer distribution within the entire tumor mass^19,20^. The SUV is influenced by many factors, and the SUVmax has been found to be unreliable and therefore is not recommended because of poor reproducibility (3% ± 11%)^21,22^. Given these controversies, researchers have suggested that volume-based variables, such as the SUVmean and metabolic tumor burden, including MTV and TLG, quantitatively reflect the metabolic activities within the entire tumor mass. The values indicate not only the intensity of FDG accumulation but also the extent of metabolic volume, thus avoiding bias due to the heterogeneity within the entire tumor.

Studies have verified the promise of ^18^F-FDG PET/CT for patients with TETs; most studies have found that FDG uptake in thymic carcinomas is significantly higher than that in thymomas^12–14,23^. The intensity of FDG uptake or the SUV is associated with the grade of the malignancy: high FDG uptake reflects the invasiveness of the malignancy or the proliferation ability in TETs^24–27^. Metabolic tumor burden and volume-dependent parameters, including the MTV and TLG, have also been reported to differentiate benign mediastinal tumors from malignant tumors, but not to correlate with the WHO classification of TETs^27^. In our study, which involved only TETs and PMLs, both the metabolic and volumetric variables were able to distinguish malignant tumors from benign tumors, and the SUVmax had the best performance and thus may be used to decrease the high rate of unnecessary thymectomies.

Ruling out PMLs from the TETs in the pre-vascular compartment of the mediastinum is crucial because PMLs are the disease most commonly misinterpreted as TETs pre-operatively. In our study, not only the SUVmax but also the volumetric variables, including the SUVmean, MTV, and TLG, were significantly different between TETs and PMLs. Among these metabolic and volumetric parameters, SUVmean had the highest sensitivity and accuracy, with a cut-off value of 6.9. The performance of the SUVmean was similar to but slightly better than that of the SUVmax. The MTV had relatively high specificity (81.5%) but lower sensitivity than the other parameters, because the TLG is the product of the MTV and SUVmean. The TLG had the same sensitivity as the SUVmax but lower specificity than the SUVmax.

Overall, all these metabolic and volumetric variables are stable, with a sensitivity and specificity of approximately 70%, but are not satisfactory for clinical use. Because both TETs and PMLs are age-dependent, we introduced age as a collaborative index. The sensitivity and specificity significantly increased to 80% and 93%, respectively, when age and the SUVmax were combined as an independent tool to differentiate TETs from PMLs. The combination of age and SUVmean resulted in higher sensitivity (83.1%) but lower specificity (88.7%) than age and the SUVmax. Both the combined tools showed much better diagnostic performance, with an AUC > 0.9. However, these results are preliminary and must be further tested before widespread clinical use.

There were several limitations to this study. First, it was a retrospective cohort study and therefore had unavoidable bias. Although other CT characteristics, such as morphology, density, enhancement manifestations, and metastasis sites/numbers can also be used to differentiate PMLs and TETs, previously published papers have discussed those points in detail. Therefore, we focused only on the effectiveness of metabolic parameters. This study would have been more comprehensive if more data were included and analyzed. Second, the ratio of subtypes in both PML and TET, such as low-risk thymomas, would affect the mean value of metabolic parameters as well as the difference between PML and TET. In addition, some types of lymphoma that are not commonly seen in the mediastinum, such as MALT, were not examined in this study. The average value of FDG uptake might have been lower if MALT had been included, because it is not FDG-avid. Third, a larger sample of patients with lesions in the pre-vascular compartment of the mediastinum must be included for better understanding of the usefulness of the metabolic tumor burden in the pre-vascular mediastinum.

## Conclusions

Our study confirmed the roles of metabolic and volumetric variables in the diagnosis and differentiation of PMLs from TETs in the pre-vascular compartment of the mediastinum. Our results have practical value in providing more accurate and reliable evaluation for differential diagnosis of thymic lesions pre-operatively. FDG PET/CT parameters, the SUVmax, SUVmean, volumetric parameters (TLG and MTV), and the combination of age and SUVmax or SUVmean could help prevent more patients from receiving unnecessary thymectomy or other invasive surgical procedures.

## Figures and Tables

**Table 1:** Demographics of patients diagnosed with primary mediastinal lymphoma and thymic epithelial tumors in this study

Pathological subtypes	*N*	Age	Age range	Male (%)
Primary mediastinal lymphoma	71 (52.2%)	30.25 ± 14.44	3–70	22 (31.0)
Classic Hodgkin lymphoma	26	26.19 ± 9.82	6–50	23.1
Diffuse large B-cell lymphoma	30	36.43 ± 14.1	15–70	30.0
T-lymphoblastic lymphoma	9	19.33 ± 16.9	3–46	55.6
Other types	6	33.33 ± 16.86	13–59	33.3
Thymic epithelial tumors	65 (47.8%)	54.23 ± 15.16	12–77	40 (61.5)
Thymoma_A	4	64.75 ± 7.41	55–73	50.0
Thymoma_AB	4	57.00 ± 9.13	46–68	50.0
Thymoma_B1	8	53.88 ± 12.51	41–76	62.5
Thymoma_B2	6	52.5 ± 21.67	16–72	66.7
Thymoma_B3	4	48.75 ± 13.3	33–64	75.0
Thymic carcinomas	39	53.77 ± 15.97	12–77	61.5
Squamous cell carcinoma	26	55.19 ± 15.48	12–76	61.5
Neuroendocrine carcinoma	8	58.25 ± 17.43	26–77	62.5
Adenocarcinoma/sarcoma	5	39.2 ± 8.58	24–45	60.0
Total	136	41.57 ± 19.12	3–77	62 (45.6)

**Table 2:** Group comparison between the primary mediastinal lymphoma and thymic epithelial tumors

	Primary mediastinal lymphoma	Thymic epithelial tumors	Group comparison	
			χ^2^	*P*
Age			55.19	< 0.001**
< 40	54	8		
≥ 40	17	57		
Sex			12.68	< 0.001**
Male	22	40		
Female	49	25		
Malignancy			33.21	< 0.001**
Malignant	71	40		
Benign	0	25		
SUVmax	16.55 ± 6.38	10.64 ± 6.16	28.18	< 0.001**
< 13.7	27	48	17.47	< 0.001**
≥ 13.7	44	17		
SUVmean	9.80 ± 3.93	6.11 ± 3.55	28.93	< 0.001**
< 8.0	29	49	16.43	< 0.001**
≥ 8.0	42	16		
MTV (cm^3^)	143.98 ± 149.93	84.99 ± 130.10	5.74	< 0.05*
< 115.8	39	53	10.9	= 0.001**
≥ 115.8	32	12		
TLG (g/mL cm^3^)	1546.1 ± 1838.69	641.78 ± 1381.45	14.55	< 0.001**
< 1113.9	42	57	13.85	< 0.001**
≥ 1113.9	29	8		

SUVmax, maximum standardized uptake value; SUVmean, mean standardized uptake value; MTV, metabolic tumor volume; TLG, total lesion glycolysis; * and ** represent significant differences of *P* < 0.05 and *P* < 0.01, respectively.

**Table 3:** Comparisons of metabolic and volumetric parameters in groups of patients with primary mediastinal lymphoma and thymic epithelial tumors

Pathological subtypes	SUVmax	SUVmean	MTV	TLG
Total	13.72 ± 6.92	8.03 ± 4.17	115.79 ± 143.37	1113.95 ± 1692.13
Primary mediastinal lymphoma	16.55 ± 6.38	9.80 ± 3.93	143.98 ± 149.93	1546.15 ± 1838.69
Classic Hodgkin lymphoma	14.05 ± 5.27	8.51 ± 3.53	73.4 ± 101.75	669.34 ± 888.29
Diffuse large B-cell lymphoma	20.81 ± 5.69	12.28 ± 3.44	180.92 ± 143.32	2327.82 ± 2082.62
T-lymphoblastic lymphoma	11.94 ± 3.39	6.87 ± 1.95	188.06 ± 134.80	1459.41 ± 1387.87
Other types	12.99 ± 5.53	7.32 ± 3.25	199.05 ± 271.13	1567.05 ± 2733.1
χ^2^, *P*	23.34, < 0.001	22.47, < 0.001	13.15, 0.004	13.96, 0.003
Thymic epithelial tumors	10.64 ± 6.16	6.11 ± 3.55	84.99 ± 130.10	641.78 ± 1381.45
Thymoma_A	6.37 ± 0.86	3.76 ± 0.52	15.65 ± 7.77	56.34 ± 24.43
Thymoma_AB	5.37 ± 2.00	3.14 ± 1.28	52.94 ± 36.31	136.72 ± 53.78
Thymoma_B1	6.16 ± 2.61	3.59 ± 1.71	39.21 ± 22.59	122.32 ± 58.77
Thymoma_B2	8.65 ± 3.85	5.07 ± 2.30	43.29 ± 21.51	210.08 ± 137.81
Thymoma_B3	9.91 ± 8.48	5.97 ± 5.37	59.60 ± 38.04	317.35 ± 253.83
Thymic carcinomas	12.91 ± 6.29	7.35 ± 3.60	113.79 ± 160.87	959.87 ± 1715.11
Squamous cell carcinoma	14.22 ± 6.68	8.25 ± 3.86	99.86 ± 121.29	1005.18 ± 1872.20
Neuroendocrine carcinoma	9.99 ± 3.94	5.86 ± 2.34	184.24 ± 278.80	1168.21 ± 1754.16
Adenocarcinoma/sarcoma	10.78 ± 5.98	5.04 ± 2.06	73.54 ± 68.25	391.46 ± 386.17
χ^2^, *P*	22.51, 0.002	23.05, 0.002	11.58, > 0.05	20.61, 0.004

SUVmax, maximum standardized uptake value; SUVmean, mean standardized uptake value; MTV, metabolic tumor volume; TLG, total lesion glycolysis.

**Table 4:** Diagnostic ability of metabolic and volumetric parameters of PET/CT in differentiating patients with primary mediastinal lymphoma versus thymic epithelial tumors

	Cut-off values	Sensitivity (%)	Specificity (%)	Accuracy (%)	AUC (95% CI)
SUVmax	12.3	70.4	70.8	70.6	0.764 (0.685–0.843)
SUVmean	6.9	76.1	69.3	72.8	0.767 (0.688–0.847)
TLG (g/mL cm^3^)	350.3	70.4	63.1	69.0	0.690 (0.599–0.780)
MTV (cm^3^)	106	46.5	81.5	66.1	0.619 (0.524–0.715)
SUVmax + TLG	/	66.2	73.2	/	0.768 (0.689–0.847)
SUVmax + age	/	80.0	93.0	/	0.908 (0.855–0.961)
SUVmean + age	/	83.1	88.7	/	0.912 (0.861–0.964)

SUVmax, maximum standardized uptake value; SUVmean, mean standardized uptake value; MTV, metabolic tumor volume; TLG, total lesion glycolysis.
